# Adolescents’ use of the built environment for physical activity

**DOI:** 10.1186/s12889-015-1596-6

**Published:** 2015-03-15

**Authors:** Nicolas M Oreskovic, James M Perrin, Alyssa I Robinson, Joseph J Locascio, Jeff Blossom, Minghua L Chen, Jonathan P Winickoff, Alison E Field, Chloe Green, Elizabeth Goodman

**Affiliations:** Massachusetts General Hospital, Boston, MA USA; Harvard Medical School, Boston, MA USA; Harvard Center for Geographic Analysis, Boston, MA USA; Boston Children’s Hospital, Boston, MA USA

**Keywords:** Physical activity, Built environment, Accelerometer, Adolescents, Global positioning system, Geographic information systems

## Abstract

**Background:**

Physical activity is a health-enhancing behavior, but few adolescents achieve the recommended levels of moderate-to-vigorous physical activity. Understanding how adolescents use different built environment spaces for physical activity and activity varies by location could help in designing effective interventions to promote moderate-to-vigorous physical activity. The objective of this study was to describe the locations where adolescents engage in physical activity and compare traditional intensity-based measures with continuous activity when describing built environment use patterns among adolescents.

**Methods:**

Eighty adolescents aged 11–14 years recruited from community health and recreation centers. Adolescents wore accelerometers (Actigraph GT3X) and global positioning system receivers (QStarz BT-Q1000XT) for two separate weeks to record their physical activity levels and locations. Accelerometer data provided a continuous measure of physical activity and intensity-based measures (sedentary time, moderate-to-vigorous physical activity). Physical activity was mapped by land-use classification (home, school, park, playground, streets & sidewalks, other) using geographic information systems and this location-based activity was assessed for both continuous and intensity-based physical activity derived from mixed-effects models which accounted for repeated measures and clustering effects within person, date, school, and town.

**Results:**

Mean daily moderate-to-vigorous physical activity was 22 minutes, mean sedentary time was 134 minutes. Moderate-to-vigorous physical activity occurred in bouts lasting up to 15 minutes. Compared to being at home, being at school, on the streets and sidewalks, in parks, and playgrounds were all associated with greater odds of being in moderate-to-vigorous physical activity and achieving higher overall activity levels. Playground use was associated with the highest physical activity level (β = 172 activity counts per minute, SE = 4, p < 0.0001) and greatest odds of being in moderate-to-vigorous physical activity (odds ratio 8.3, 95% confidence interval 4.8-14.2).

**Conclusion:**

Adolescents were more likely to engage in physical activity, and achieved their highest physical activity levels, when using built environments located outdoors. Novel objective methods for determining physical activity can provide insight into adolescents’ spatial physical activity patterns, which could help guide physical activity interventions. Promoting zoning and health policies that encourage the design and regular use of outdoor spaces may offer another promising opportunity for increasing adolescent physical activity.

## Background

Physical activity has many associated health benefits, decreases risk of obesity, and independently decreases morbidity and mortality. There is mounting evidence on the association between certain built environments, particularly outdoor spaces such as parks and playgrounds, and higher physical activity levels [1-5]. Investigators are beginning to use Global Positioning System (GPS) receivers along with accelerometers to better understand how youth use the built environment [6]. However, there are three major gaps in this field which hinder optimizing adolescents’ use of the built environment. First, no standardized method exists for measuring physical activity in the built environment. Most prior work in this emerging area has assessed physical activity using discrete activity levels, such as sedentary and moderate-to-vigorous physical activity (MVPA) [3,7-9], with continuous physical activity measures used less often [10], and little is known about patterns and variations in built environment use over time [2]. Second, although national adult health guidelines recommend physical activity bouts of at least 10 consecutive minutes [11-13], pediatric guidelines do not address bout length, recommending only that children achieve a minimum of 60 minutes of MVPA per day [13]. Third, while strategies for assessing and analyzing use of the built environment for physical activity are beginning to offer a better understanding of how much time youth spend using various built environment attributes and how activity levels vary by location [7,8], questions remain about the most useful way to analyze use of the built environment for physical activity.

In this study, we sought to address these gaps in the current knowledge base by first describing the different locations where adolescents spend time as well as the patterning of adolescent physical activity, and second, by comparing the use of a traditional intensity-based physical activity (MVPA) measure with continuous physical activity data when analyzing use of the built environment among youth.

## Methods

### Participants

From April 2011-April 2012, we recruited a convenience sample of 96 non-Hispanic white, non-Hispanic black, and Hispanic 11–14 year olds who sought care at a community health and/or attended a community recreation center living in three surrounding predominantly urban middle- and low-income communities in the greater Boston area. Age eligible subjects without physical impairments limiting ambulation were recruited sequentially during the study enrollment period. Signed parental informed consent along with signed child assent was obtained from each family. For inclusion in this study, subjects had to have accelerometer and GPS data available from at least one of the two measurement periods. During the first measurement period, five subjects dropped out of the study, two were lost to follow up, and eight provided inadequate data. During the second measurement period, four subjects provided insufficient data, sixteen subjects declined participation, two subjects relocated, and twelve subjects were unable to be contacted. A total of 80 (83.3%) participants completed the study with sufficient data and were included in the final study sample. The Partners HealthCare Institutional Review Board approved the study.

### Data collection and measures

A researcher provided each subject with a hip-worn belt equipped with an accelerometer (GT3X; ActiGraph LLC) to record physical activity and a GPS receiving unit (QStarz BT-Q1000XT) to record location. Accelerometer and GPS devices were both set to record at 30 second intervals, with their internal clocks synchronized. Subjects were asked to wear the belt at all times during waking non-water activity hours for at least 5 weekdays and 2 weekend days and to recharge their GPS overnight in two separate seasons, one warm (September through mid-November, April through June) and one cold (mid-December through March), to account for known seasonal variations in physical activity levels and built environment use [7]. Study staff recorded the temperature (high, low, average; recorded as Fahrenheit to the nearest whole unit) and weather condition (sun; overcast; rain; snow) on each study day.

At entry, trained research staff measured height and weight using a stadiometer (SECA) and digital scale (LifeSource MD) with participants wearing indoor clothing, pockets emptied, and shoes removed. Measurements were taken in duplicate then averaged, from which body mass index (BMI) was calculated (kg/m^2^), then used to determine participants’ weight status (healthy weight, overweight, obese) according to CDC age-and sex-specific percentile cut-offs [14]. Self-reported age (date of birth), sex, and race/ethnicity were obtained at baseline, along with highest parent-reported level of parental education.

### Data processing

#### Data merging and processing

Study personnel reviewed GPS and accelerometer output to ensure each subject had at least 2 hours of daily time-matched data, on at least 2 week days and 1 weekend day, with accelerometer data having at least 10% non-zero epochs per hour [15]. Datasets that met minimum inclusion criteria were then cleaned to exclude days and/or hours of non-wear as defined by the validation criteria above (e.g. hours with <10% non-zero epochs, days with <2 hours valid time-matched data). Data occurring during overnight (12 AM-5 AM) hours were removed, along with accelerometer datapoint(s) at the start of a day without a matching GPS datapoint. Furthermore, to avoid misclassification of imputed GPS data due to GPS malfunction (signal loss, excessive cold- and warm-start up times, jitter, drift) or battery depletion, protracted missing GPS data (>2 hours) during non-school hours were also removed. Review of data revealed signal loss occurring frequently in larger buildings, most prominently schools, and infrequently in residential buildings. GPS data missing for > 2 hours during school hours was thus imputed, as this was felt to represent appropriate indoor signal loss. Accelerometer and GPS data were joined according to the date and time information in each unit based on a prior published software program written in 2011 [7]. For accelerometer datapoints without a corresponding GPS point, the missing latitude and longitude were imputed using the last previously recorded values. The joined data were collapsed into 1 minute epochs for all data analyses to align with recent GPS-accelerometer studies and physical activity guidelines [2,3,7,10,16].

#### Land use classifications

Each subject’s home address and school and all joined data were geo-classified using geographic information system (GIS) (ArcGIS 9.2). Each GPS datapoint was assigned one of six distinct land-use classifications: home, school, park, playground, street/sidewalk, or other [7] using the Commonwealth of Massachusetts’ Office of Geographic Information (MassGIS) Land Use 2005 GIS layer. Location data were further categorized as indoor (home and school), outdoor (park, playground, street/sidewalk), or other. MassGIS classifies land use into 40 different types as observed from 50 cm pixel resolution color orthoimagery, with playground including areas built specifically for public recreation (soccer, football, and baseball fields, golf courses) and park including green spaces and open land (forest, town green, beaches, open park and public space). MassGIS land-use assignments were verified using 30 cm pixel resolution 2008 color orthoimagery. Forty meter buffers around the center of the subject’s house and school building perimeter, and a hierarchical land-use classification system was used to account for inherent GPS error and reduce possible misclassification.

#### Physical activity data

Accelerometers provided activity counts per minute. Due to skewness, count data were log transformed for analysis as continuous data. In addition to using continuous physical activity data for analyses, to relate the findings to current physical activity guidelines and compare our findings to the literature, we also classified activity data into intensity-based categories, with <100 counts per minute as sedentary and ≥2296 counts per minute (corresponding to 4 metabolic equivalents) as moderate-to-vigorous physical activity (MVPA) [17].

### Analysis

Univariate analyses included deriving mean daily minutes of total time, MVPA, and sedentary time that youth spent in each land-use category. We also plotted physical activity counts per minute versus location data separately by person-date and visually inspected the plots for patterns in physical activity and variations in land-use. Using these longitudinal plots, we compared total daily energy expenditure (Kcal) from MVPA to energy expenditure achieved from all daily activity levels below MVPA threshold [18].

Bivariate analyses tested for associations of physical activity counts and minutes of each physical activity category (sedentary and MVPA) by grouped land-use categories (indoor, outdoor, other). Nonparametric tests (Mann Whitney U and Kruskal Wallis) were used due to skewed distributions. Because these are time series data, autocorrelation (where activity at one minute is not independent from activity at previous minutes) is a concern. When autocorrelation is present, it can bias the estimates. To account for this, time series analysis (SAS Arima procedure) was used to test for autocorrelation up to the fifteenth order separately for each person-date and removed prior to subsequent longitudinal analyses. Time series analyses by person-date also allowed us to test for bout length. Finally, we performed multivariable modeling adjusting for potential confounders (age, sex, race/ethnicity, weight status, parental education, valid hours of combined data, season, temperature, and weather) to assess the relationship between land-use categories and physical activity. We first ran longitudinal mixed effects models that accounted for repeated measurements and clustering effects with continuous physical activity (log counts per minute) as the dependent variable, built environment variables as fixed predictors, with polynomial contrasts of time as random effects, and the covariates noted above. A backward elimination (with cutoff of p ≤ 0.01) algorithm was applied. We performed interaction analyses for location and weather to explore possible effect modification of activity by weather. We then used generalized estimating equation logistic regression models to test the association of land-use categories and intensity-based measures of physical activity, accounting for repeated measures and clustering effects, adjusting for potential confounders. Dependent variables for these logistic regression analyses were whether a minute was classified as MVPA (yes/no) or sedentary (yes/no), respectively.

## Results

Table 1 describes the study sample. The mean age of participants was 12.6 years, with 44% male, 40% white, 23% black, 36% Hispanic or Latino, and 49% overweight or obese. The majority of adolescents resided in the town where the community health and recreation center were located. Most (80%) adolescents lived in households where one or more parent had a high school education or higher, with 6% of parents reporting less than a high school education, and 14% missing or not reporting parental education.

**Table 1:** **Demographic characteristics of study participants (N = 80)**

**Characteristic**	**n (%) or Mean ± SD**
Age, yr	12.6 ± 1.1
Male	35 (43.8)
Race	
White	32 (40.0)
Black	18 (22.5)
Hispanic	29 (36.3)
Unknown	1 (1.2)
BMI	
<85^th^ percentile	41 (51.2)
85^th^-95^th^ percentile	18 (22.5)
≥95^th^ percentile	21 (26.3)
Town	
Town 1	66 (82.5)
Town 2	8 (10.0)
Town 3	6 (7.5)

A total of 564,528 minutes of combined GPS accelerometer data were included for analysis in the final dataset, averaging 9.8 hours per study day. An average of 12.1 days (range:3–26) days of combined data were collected per subject, with a mean of 7.8 (range:3–14) days in the first season (n = 79) in which data were collected and 8.4 (range:3–18) days in the second season (n = 45).

**Figure 1:**
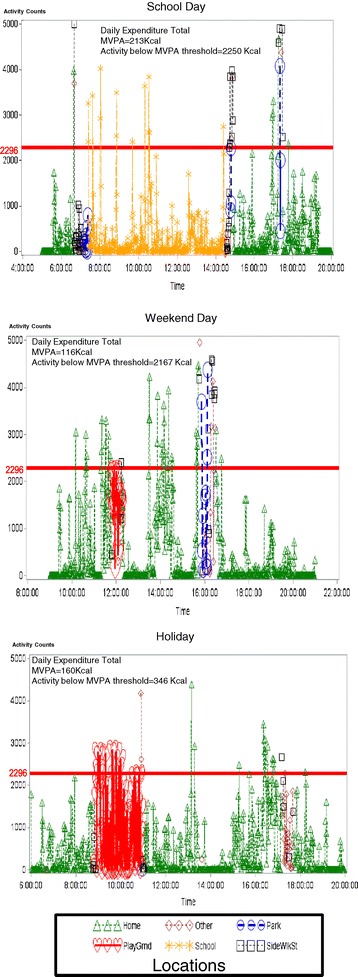
**Illustrative examples of longitudinal plotting of a subject’s daily physical activity data versus time with different symbols (triangle, diamond, circle, heart, star, square) representing various locations.** *Activity counts >2296 constitute moderate-to-vigorous physical activity (MVPA). **Physical activity data presented over time in 1 minute intervals.

An average of 277 minutes of data were collected per adolescent per day at home, along with 296 minutes at school, 45 minutes in streets and sidewalks, 25 minutes at playgrounds, 17 minutes at parks, and 99 minutes in all other locations (p < 0.001 for indoor vs. outdoor vs. other locations). Visual inspection of the longitudinally plotted data revealed substantial variation of youth physical activity over time, both within and between land-use categories. Figure 1 provides an illustrative example of a single subject’s activity levels across time of day for three different days. The adolescent’s location and physical activity intensity in each different location is noted to vary by type of day (school vs. non-school day) and time of day. The figure also reports the total daily energy expenditures calculated for each day, with daily MVPA tallies ranging from one half to one twentieth the daily energy expenditure approximations derived from activity data below the MVPA cut-off.

**Figure 2:**
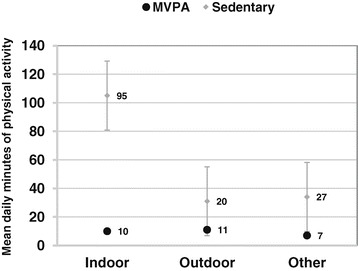
**Daily minutes of physical activity by intensity level and location.** MVPA, moderate-to-vigorous physical activity.

Overall, physical activity levels rarely reached the MVPA threshold; only 3.4% of all minutes collected were categorized as moderate-to-vigorous physical activity. Bouts of MVPA lasted up to 15 minutes. A mean of 21.7 minutes (SD 15.8; range:6.8-124.3) of MVPA were collected per day per participant. Adolescents achieved similar minutes per day of MVPA in both indoor and outdoor locations. Sedentary time was far more prevalent than MVPA. A mean of 133.6 (SD 34.8; range:37.1-227.0) minutes of sedentary time were collected per day per participant. Unlike mean MVPA, mean sedentary time (SD) was considerably greater in indoor than outdoor or other locations [μ_indoor_ = 95(29) vs. μ_outdoor_ = 20(14) vs. μ_other_ = 27(20), p < 0.0001] (Figure 2).

Among indoor locations, adolescents achieved more minutes of both MVPA and sedentary time in schools than at home (median_school_ daily mean MPVA = 8 [Interquartile Range (IQR):5–12] minutes vs. median_home_ = 4 [IQR:2–8], p < 0.001; median _school_ daily mean sedentary minutes = 87 [IQR:63–110] vs. median_home=_50 [IQR:40–69], p < 0.0001). In outdoor spaces, more minutes of MVPA were recorded on streets and sidewalks than in playgrounds or parks (median_streets and sidewalks_ daily mean MVPA = 5 [IQR:3–9] vs. median_playgrounds_ = 3 [IQR:1–6] vs. median _parks=_2 [IQR:1–4], p = 0.02), though total MVPA levels and differences were small. Streets and sidewalks also accounted for the greatest amount of daily outdoor sedentary time (p = 0.003).

### Adjusted associations of physical activity with the built environment

In longitudinal mixed effects models using continuous physical activity counts rather than MVPA/sedentary, the built environment showed broad associations with physical activity (Table 2). Compared to being at home, all other locations, including all outdoor land-use categories, were associated with higher recorded physical activity counts. Being in a playground was associated with the highest levels of physical activity, with each additional minute of playground use resulting, on average, in an additional 172 counts per minute of activity. Boys averaged 34 more counts per minute than girls. Temperature significantly predicted physical activity level, while weather modified physical activity across all locations with snow having the greatest influence on activity. During snow days, adolescents had increased activity at playgrounds and parks compared to other locations or other weather conditions, while adolescents had increased activity on streets and sidewalks during sunny and overcast weather compared to rain and snowy days (Table 3).

**Table 2:** **Determinants of adolescent physical activity, using continuous physical activity (counts per minute) data**

	**β** ^**a**^	**SE**	***p*** **-value** ^**b**^	**DF**
**Location**			<0.0001	5
Home	Ref	-	-	-
School	0.42	0.02	<0.0001	-
Street/Sidewalk	1.47	0.02	<0.0001	-
Playground	1.72	0.04	<0.0001	-
Park	1.13	0.04	<0.0001	-
Other	0.80	0.02	<0.0001	-
Weather	-	-	0.6	3
Location * Weather	-	-	<0.0001	15
Temperature	0.007	0.002	0.0002	1
Gender			<0.0001	1
Female	Ref			-
Male	0.34	0.05	<0.0001	-

Ref, reference group; SE, standard error; DF, degree of freedom. Model includes 564,448 minutes of combined location-activity data among 80 subjects, and is adjusted for subject, age, sex, weight category, date, town, temperature, weather, and location-weather interactions, as well as time sequences (polynomial time terms remained significant up to time^9^, p < 0.0001) including valid hours per day of combined data to account for potential temporal effects of physical activity. The model AIC = 2,590,976.

^a^Reported β estimates for location variables are the log of activity counts (activity counts = ß × 100); β estimates for covariates are partial regression coefficients.

^b^Longitudinal mixed effects.

Location*Weather indicates interaction term for Location and Weather.

**Table 3:** **Effect of location and weather on adolescent physical activity (n = 564,449 minutes of combined activity and location data)**

	**Rain**	**Snow**	**Overcast**	**Sun**
Home	2.06 (1.91-2.21)	2.42 (1.80-3.03)	2.20 (2.12-2.28)	2.23 (2.15-2.29)
School	3.16 (3.01-3.32)	3.13 (2.46-3.81)	2.71 (2.63-2.79)	2.64 (2.57-2.72)
Street/Sidewalk	3.28 (3.12-3.44)	2.02 (1.35-2.69)	3.69 (3.61-3.78)	3.68 (3.59-3.76)
Playground	3.94 (3.74-4.14)	7.25 (5.23-9.26)	3.89 (3.78-4.00)	3.98 (3.88-4.08)
Park	3.29 (3.07-3.52)	5.33 (2.56-8.11)	3.29 (3.18-3.41)	3.41 (3.30-3.52)
Other	3.19 (3.04-3.34)	2.67 (2.03-3.29)	3.05 (2.96-3.13)	3.05 (2.97-3.12)

Values reported are adjusted least square means for the log of activity counts, along with 95% confidence intervals in parentheses. Models are adjusted for subject, age, sex, weight category, date, temperature, weather, and location-weather interactions, as well as time sequences (polynomial time terms remained significant up to time^8^, p < 0.0001) to account for potential temporal effects of physical activity.

Multivariable analyses of physical activity separated into MVPA and sedentary time showed all land-use types predicting higher odds of a minute being in MVPA compared to a minute at home (Table 4). Adolescents had nearly 7 times the odds of a minute being in MVPA on streets and sidewalks, over 8 times the odds of a minute being in MVPA in playgrounds, and 5 times the odds of a minute being in MVPA in parks compared to being at home. Black compared to white adolescents had increased odds of a minute being in MVPA, as did boys compared with girls. All land-use types were associated with higher odds of one minute being in sedentary time compared to one minute spent at home, with all land-uses having less than twice the odds.

**Table 4:** **Predictors of a minute of adolescent time being in moderate-to-vigorous or sedentary time**

	**MVPA**			**Sedentary Time**		
**Predictors**	**OR**	**95% CI**	***p*** **-value** ^**a**^	**OR**	**95% CI**	***p*** **-value** ^**a**^
Location						
Home	Ref			Ref		
School	1.73	1.37-2.19	<0.0001	1.64	1.51-1.79	<0.0001
Street/Sidewalk	6.75	4.72-9.64	<0.0001	1.82	1.61-2.05	<0.0001
Playground	8.26	4.81-14.19	<0.0001	1.19	0.96-1.48	0.12
Park	4.86	3.03-7.78	<0.0001	1.25	1.07-1.46	0.006
Other	2.94	2.22-3.89	<0.0001	1.35	1.26-1.45	<0.0001
Race/Ethnicity						
White	Ref			Ref		
Black	1.57	1.03-2.39	0.03	0.88	0.73-1.04	0.14
Hispanic	1.10	0.75-1.61	0.64	0.95	0.81-1.10	0.48
Gender						
Female	Ref			Ref		
Male	1.57	1.19-2.06	0.001	1.02	0.91-1.13	0.77

MVPA, moderate-to-vigorous physical activity; OR, odds ratio; Ref, reference group. Models are adjusted for age, weight status, town, temperature, and weather.

^a^Generalized estimating equation logistic regression.

## Discussion

In this study which used GPS and accelerometer data, we found that intensity-based and continuous physical activity analysis methods revealed a statistically significant role for the built environment in adolescent physical activity. This study is among the first to compare intensity-based to continuous physical activity data when assessing physical activity by location in youth. Both analysis methods identified outdoor spaces as superior to indoor spaces for promoting physical activity. Playgrounds were the location with the greatest predictive value for being in MVPA, and were also the location with the highest recorded physical activity levels. We also plotted physical activity and built environment use patterns over time to delineate the temporal and sporadic nature of adolescent physical activity.

Though schools have received much policy and research attention as desirable venues for promoting physical activity, similar to a study in English children, we found physical activity levels among adolescents to be higher in outdoor than indoor spaces [2]. These findings, along with our findings that adolescents obtained only about one third the daily recommended levels of physical activity, would argue for increased efforts by urban planners and public officials to create more outdoor environments which are ‘activity friendly’ and will attract youth outdoors.

Using intensity-based (MVPA and sedentary time) and continuous physical activity data, we confirmed previous research identifying parks and playgrounds as spaces that promote physical activity [4,5,19]. However, prior studies measuring physical activity at playgrounds have tended either to assess only specific facilities or not simultaneously record activity occurring at other locations to allow for direct comparison of activity levels by land-use category. A recent similar study of younger children found that only 2% of children’s total daily physical activity occurred at parks or playgrounds. This study did not disassociate the physical activity time spent in parks, which have been the most well studied location [3,20], from that spent in playgrounds, nor did it compare activity intensity in parks and playgrounds to activity levels in other land-uses.[10] Though we found that both parks and playgrounds were important locations for obtaining physical activity, playgrounds, streets and sidewalks were the most likely locations for recorded physical activity in adolescents, more so even than parks. This finding was consistent using both continuous and intensity-based physical activity analyses methods. The planning literature has long identified the importance of a well-connected street system to encourage pedestrian activity [21,22], yet less is known about the potential benefits of street and sidewalk systems as direct promoters of higher level physical activity. We found all land-use categories to be associated with sedentary time relative to time spent at home, yet the odds for being sedentary were considerably lower and more uniform than the odds for being in MVPA in these locations. This suggests that sedentary time may be more of a common daily function that occurs throughout the day and that has less of a potential to be impacted by urban form or location than higher-level physical activities which showed greater variations by land-use type.

To our knowledge, this is one of the first built environment studies to test for bout lengths. We found that in a free-living scenario, when adolescent physical activity was recorded throughout the entire day and over all locations, physical activity occurred in bouts lasting up to fifteen minutes. Further research should assess whether bouts vary by time of day, activity, and location, given our observed variations in activity levels. The health benefits of prolonged versus intermittent physical activity also remain unclear [23,24]. Adult physical activity guidelines are based on 10 minutes bouts [13], and prior pediatric studies looking at physical activity bout lengths have used 5 minute bins to study associations between physical activity and health outcomes [25,26]. One study assessing weight status outcomes found longer bouts of MVPA to protect against risk of overweight [25], while another study reported equal cardiovascular benefits from sporadic (<5 consecutive minutes) versus consecutive physical activity [26]. Our finding that physical activity occurs in bouts lasting three times longer than the bout estimate used in prior pediatric health outcome studies provides useful information for the design of future adolescent physical activity interventions.

We also found that using traditional measures for quantifying physical activity (summing total minutes of daily MVPA) grossly undervalues the relative benefits adolescents derive from different built environments, by undercalculating caloric expenditure by as much as 90 percent. Adolescents in this study achieved on average the greatest total daily MVPA in schools, a mean daily MVPA of approximately 8 minutes, due to the large total daily time adolescents spent at school. However, schools were a location with generally low recorded activity levels, second only to the level recorded at home, and when physical activity was adjusted for total time spent in each location, school time was associated with low odds of being in MVPA. Comparing energy expenditure calculations derived using continuous activity data with energy expenditure calculations obtained using intensity-based activity data illustrate another way in which using MVPA to assess the health benefits of physical activity likely substantially underestimates the actual health benefits adolescents accrue from their total daily physical activity. In measuring adolescent physical activity with traditional intensity-based methods, one misses a large degree of daily activity below the moderate activity threshold with known health benefits. In addition, daily MVPA minutes may not provide actionable information, as these minutes may be separated throughout the day and may not occur in the same location. Future pediatric physical activity and obesity interventions aimed at increasing energy expenditure might consider targeting physical activity in the ranges and locations where it is most common and achievable, rather than focusing primarily on MVPA.

Our study has limitations. Subjects are from one metropolitan region and findings may not be generalizable to other populations. Subjects were not randomly selected and our sample may differ from a sample that is randomly selected. Our subjects were predominantly from one town, and while this mirrors the populations attending the community health and recreation centers, the possibility of systematic bias exists. Validity criteria for combined data were set at 2 hours to minimize data loss given known difficulties in obtaining simultaneous GPS and accelerometer data, including GPS signal loss, start-up time, jitter and drift, and battery depletion. To account for this low threshold and adjust for days with more limited physical activity, we included a covariate for valid hours of day, a commonly used method in accelerometry studies. We imputed missing GPS data to re-capture data loss due to indoor signal loss, a well-known limitation of using satellite signals. Despite taking steps to avoid location misclassification, including only imputing data falling within the same day and limiting the amount of consecutive imputed data, misclassification bias remains possible. In sub-analyses we found location misclassification to be minimal (<2%). Though we identified location and temporal patterns in physical activity, we did not test for spatial autocorrelation [27,28], nor did we include health outcomes. We identified fifteen minutes as the ceiling for continuous physical activity among adolescents. However, without further information on the distribution of adolescent bout lengths, it is difficult to interpret the relative frequency of these findings and caution should be employed when attempting to use this finding to counsel adolescents on activity or in formulating physical activity recommendations.

## Conclusion

An objective assessment of adolescent physical activity patterns highlighted the importance of outdoor spaces among the cohort participating in this study. Knowledge about the locations and patterns of physical activity among targeted populations can help guide physical activity interventions and the design of outdoor spaces, to ensure that location, duration and intensity of physical activity are taken into consideration. Targeting activity interventions for adolescents solely in the MVPA range may represent a lost opportunity for physical activity promotion.

## Abbreviations

BMI: Body mass index
MVPA: Moderate-to-vigorous physical activity
GPS: Global positioning system
GIS: Geographic information systems
